# Green Microwave-Assisted Combustion Synthesis of Zinc Oxide Nanoparticles with *Citrullus colocynthis* (L.) Schrad: Characterization and Biomedical Applications

**DOI:** 10.3390/molecules22020301

**Published:** 2017-02-16

**Authors:** Susan Azizi, Rosfarizan Mohamad, Mahnaz Mahdavi Shahri

**Affiliations:** 1 Department of Bioprocess Technology, Faculty of Biotechnology and Biomolecular Sciences, Universiti Putra Malaysia, UPM Serdang, Selangor 43400, Malaysia; 2 Laboratory of Biopolymer and Derivatives, Institute of Tropical Forestry and Forest Products, Universiti Putra Malaysia, UPM Serdang, Selangor 43400, Malaysia; 3 Department of Chemistry, Shiraz Branch, Islamic Azad University, Shiraz 74731-71987, Iran; mahnaz.chem@gmail.com

**Keywords:** ZnO nanoparticles, Combustion method, green synthesis, *Citrullus colocynthis*, antimicrobial, antioxidant

## Abstract

In this paper, a green microwave-assisted combustion approach to synthesize ZnO-NPs using zinc nitrate and *Citrullus colocynthis* (L.) Schrad (fruit, seed and pulp) extracts as bio-fuels is reported. The structure, optical, and colloidal properties of the synthesized ZnO-NP samples were studied. Results illustrate that the morphology and particle size of the ZnO samples are different and depend on the bio-fuel. The XRD results revealed that hexagonal wurtzite ZnO-NPs with mean particle size of 27–85 nm were produced by different bio-fuels. The optical band gap was increased from 3.25 to 3.40 eV with the decreasing of particle size. FTIR results showed some differences in the surface structures of the as-synthesized ZnO-NP samples. This led to differences in the zeta potential, hydrodynamic size, and more significantly, antioxidant activity through scavenging of 1, 1-Diphenyl-2-picrylhydrazyl (DPPH) free radicals. In in vitro cytotoxicity studies on 3T3 cells, a dose dependent toxicity with non-toxic effect of concentration below 0.26 mg/mL was shown for ZnO-NP samples. Furthermore, the as-synthesized ZnO-NPs inhibited the growth of medically significant pathogenic gram-positive (*Bacillus subtilis* and Methicillin-resistant *Staphylococcus aurous*) and gram-negative (*Peseudomonas aeruginosa* and *Escherichia coli*) bacteria. This study provides a simple, green and efficient approach to produce ZnO nanoparticles for various applications.

## 1. Introduction

**Figure 1:**
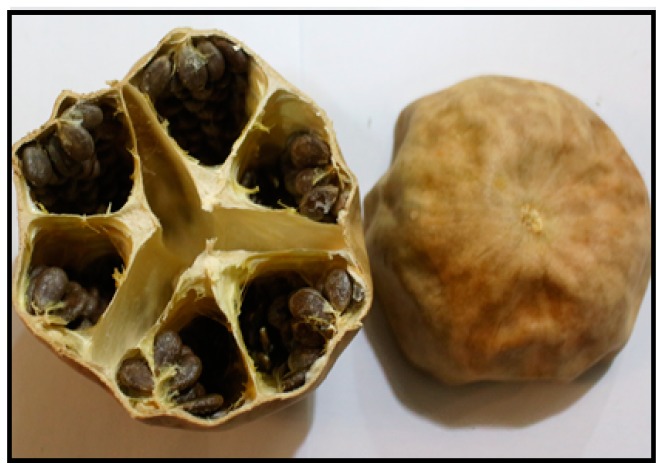
Photograph of fruits of *C. colocynthis.*

Zinc oxide nanoparticle as a non-toxic, low-cost, and non-hygroscopic metal oxide is very economical and safe polar inorganic crystalline material which has extensive applications in different areas. Nano-sized ZnO particles are an important metal oxide material which has a wide direct band gap (3.37 eV) and large exciton binding energy (60 meV) at room temperature [1,2]. The ZnO-NPs has been broadly explored for applications in catalysis [3], gas sensing [4], cosmetics [5], drug delivery [6], and solar cells [7]. There are numerous researches available on the synthesis of ZnO-NPs using various chemical and physical methods such as precipitation [8], solvothermal and hydrothermal synthesis [9,10], laser ablation [11], sonochemical [12], sol-gel [13] and flame spray synthesis [14] approaches. However, most of these synthetic methods require costly equipment and need to be operated under very severe conditions. In comparison with the mentioned techniques, combustion route has more advantages, such as simple process, fast, low cost, high purity and homogeneous products [15,16]. The method is interesting for the synthesis of multi-component oxide materials in a short time with high surface area products and highly pure crystalline particle size at low temperature [17,18]. The microwave-assisted combustion approach has been extensively used to produce various metal oxide materials such as Y_2_O_3_ [19], TiO_2_ [20], CeO_2_ [21] and ZnO [22]. However in most researches, chemical compounds have been used as fuel to synthesize metal oxide materials which may restricts their biomedical applications. To overcome this limitation, we applied a green microwave-assisted combustion approach to synthesize ZnO-NPs using fruit, seed and pulp extracts obtained from *Citrullus colocynthis* (*C. colocynthis*) plant as bio-fuels. Green synthesis is more eco-friendly with less toxic effects in comparison with chemical processes [23]. The *C. colocynthis* a valuable cucurbit plant, broadly distributed in the tropical areas of the world, has many pharmaceutical and nutraceutical values [24]. The fruits possess a soft, white spongy pulp which is filled with many smooth, compressed and oval-shaped seeds (Figure 1). Based on the previous studies, *C. colocynthis* has shown very effective antibacterial, anti-inflammatory, antioxidant and anticancer activities [25]. The therapeutic effects of *C. colocynthis* are usually attributed to its polyphenolic compounds such as *isosaponarin*, *isovitexin* and *isoorientin 3-O-methyl ether* [26,27]. These compounds with medicinal effects have the potential to be absorbed on the surface of nanoparticles during the synthesis process. Therefore, stable ZnO nanoparticles synthesized with *C. colocynthis* could be extremely suitable for drug delivery, gene delivery and biosensor applications, where there is a direct contact of these nanoparticles with blood. This study attempts to exploit *C. colocynthis* extract as a bio-fuel for the synthesis of ZnO-NPs for biomedical applications.

## 2. Results and Discussion

Zinc oxide nanoparticles using combustion process can be produced through the combination of zinc nitrates in an aqueous solution with a fuel. In the experiments, fruit, seed and pulp extracts of *C. colocynths* were suitable fuels because they contain various compounds such as Cucurbitacins, glycosides, flavonoids and phenolic acids [28,29] that can act as complexing agents of the zinc ion in the solution, as well as contributing as fuel for the synthesis of ZnO nanoparticles.

During the formation of ZnO nanoparticles an exothermic reaction takes place between oxidizing and reducing agent. Nitrate could act as an oxidizer agent and fuels could act as reducing agents for the formation of ZnO nanoparticles [30,31,32].

**Scheme 1:**
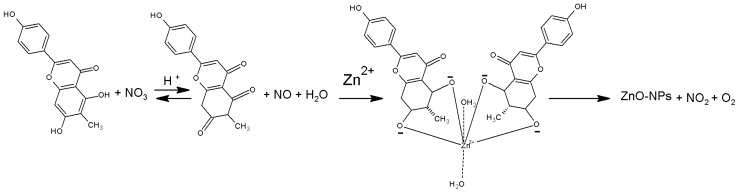
The possible mechanism for formation of ZnO-NPs with *Isovitexin* as a flavone.

The possible mechanism for formation of ZnO-NPs is as below: The nitrate group is a stronger oxidizer and can oxidize hydroxyl groups present in biomolecules to carbonyl groups and simultaneously Zn^2+^ ions form a complex compound inside the nanoscopic templates of metabolites through transfer of the π electrons from the carbonyl groups to the transition metal–dative coordinate bonding and finally, the ZnO-NPs formed by thermal decomposition of Zn^2+^ complex (Scheme 1).

### 2.1. Structural and Morphology Characterization of ZnO-NPs

**Figure 2:**
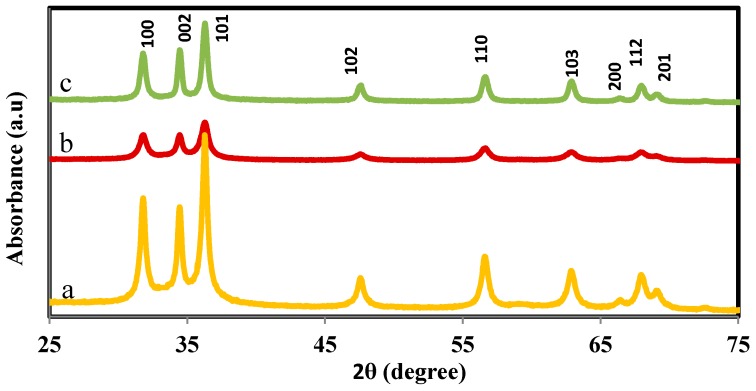
The XRD patterns of (**a**) ZnO fruit (ZnO-F), (**b**) ZnO seed (ZnO-S) and (**c**) ZnO pulp (ZnO-P) nanoparticles.

Figure 2 shows the XRD patterns of synthesized ZnO-NPs. All the recorded peaks of the (100), (002), (101), (102), (110), (103), (200), (112) and (201) can be indexed to reflection lines of hexagonal wurtzite ZnO (JCPDS 36-1451). The widening of the diffraction peaks indicates that the crystalline size of obtained particles is in nanoscale range.

The wurtzite lattice parameters, such as the values of d, the distance between adjacent planes in the Miller indices (*h k l*); lattice constants a, b, and c, interplanar angle (the angle φ between the planes (*h*_1_ *k*_1_ *l*_1_), of spacing d_1_ and the plane (*h*_2_ *k*_2_ *l*_2_) of spacing *d*_2_), and unit cell volumes (V), are calculated from the lattice geometry [33]. It was observed that there was small change in the lattice parameters of nanoparticles produced by fruit, seed and pulp extracts. The variation in lattice parameters can be ascribed to the change of particle size and quantum size effects [34]. The lattice parameters of the synthesized ZnO-NPs are summarized in Table 1.

The average crystallite size calculated using Scherrer equation D = $\frac{0.89\lambda }{\beta cos\theta }$ with the width of the (002) and (101) peaks are found to be 27 ± 2 nm, 64 ± 2 nm, and 85 ± 2 nm for ZnO prepared with seed, pulp and fruit extract, respectively.

**Figure 3:**
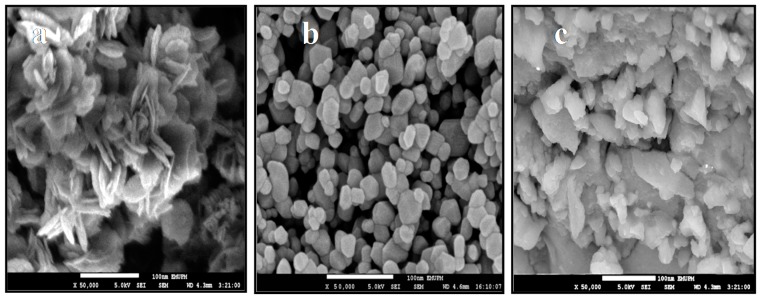
FESEM images of: (**a**) ZnO-F; (**b**) ZnO-S and (**c**) ZnO-P nanoparticles.

The morphology, size, and structure of the ZnO samples were investigated through FESEM, and TEM. As shown in Figure 3a the large quantity of flower shaped nanostructures, 85–100 nm in size produced with fruit extract. The floral nanostructures resulted from the assemblage of numerous of ZnO nanoflakes, which were created from a single axis. In ZnO-S (Figure 3b), the hexagonal nanostructures, 20–35 nm in size were generated. It is interesting to mention that the flower-like and hexagonal nanostructure produced with fruit and seed changed entirely into a blocky morphology in the ZnO sample prepared by pulp, as presented in Figure 3c. The dimensions of these irregular block-shaped nanoparticles ranged from 30 to 80 nm. The results demonstrate that bio-fuel functions as a structure-directing agent, greatly affecting the anisotropic development of ZnO in different structures of flower-shaped, hexagonal and block-like morphology.

The difference in morphology and particle size using different fuels are directly associated to the number of moles of combustion gases escaped during process. The gases disrupt bulky clusters and make holes between particles. In fact, the clusters are broken in conditions of more generating of gaseous products and in these conditions high heat is released from the system, hindering particle development [35].

**Figure 4:**
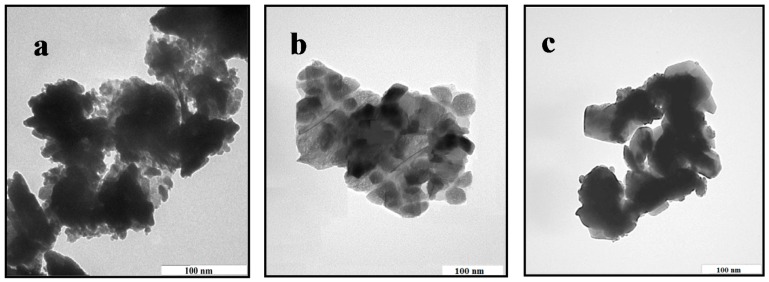
TEM images of: (**a**) ZnO-F; (**b**) ZnO-S and (**c**) ZnO-P nanoparticles.

Further structural characterization of the ZnO nanostructures was performed by TEM. Figure 4a displays the TEM image of the flower-shaped ZnO, which is accordance with the FESEM observations (Figure 3a). ZnO with hexagonal and some irregular structures are clearly seen in the TEM image of ZnO-S, as shown in Figure 4b. The TEM image of ZnO-P shown in Figure 4c proves that the shape of the nanoparticles is a combination of irregular polygons. This outcome is consistent with the FESEM result (Figure 3c)

**Figure 5:**
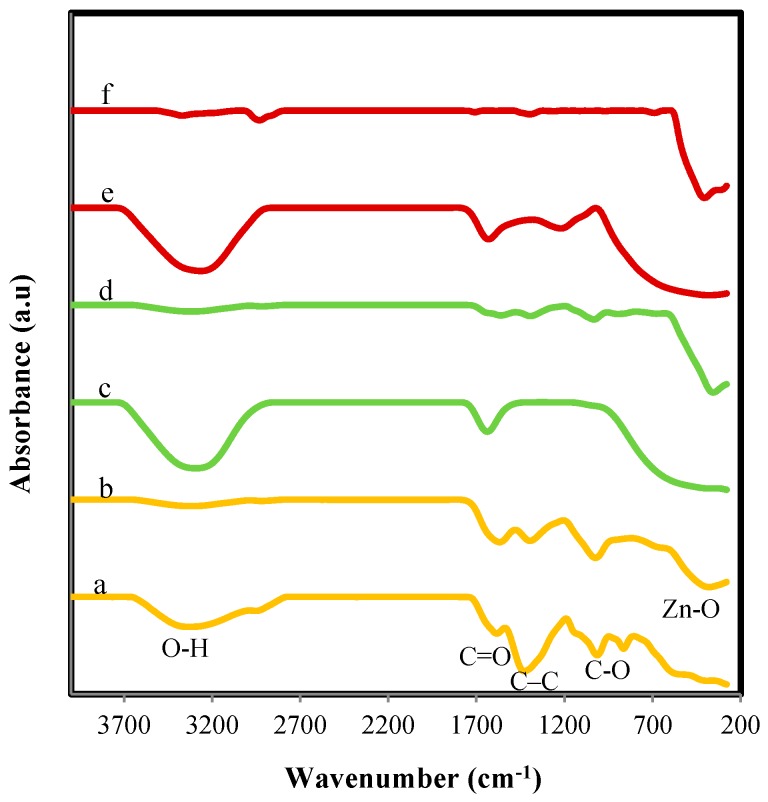
FTIR spectra of (**a**) fruit extract; (**b**) ZnO-F; (**c**) pulp extract; (**d**) ZnO-P; (**e**) seed extract and (**f**) ZnO-S.

FTIR spectra illustrate the functional groups of as-synthesized ZnO-NPs and extracts as shown in Figure 5a–f. The broad absorption band is observed in 3352 cm^−1^ can be ascribed to O-H stretching and H-bonded in glycosides, alcohol or phenol groups. The absorption peak is observed at 1630 cm^−1^ corresponding to the C=O bands. The peak observed at 1420 cm^−1^ attribute to C-C stretching in aromatic groups. The absorption bands in the range of 390–350 cm^−1^ in the spectra of synthesized nanoparticles correspond to the Zn-O. FT-IR spectra clearly show the absorption bands of O-H stretching and C=O after formation of ZnO-NPs reduced indicating the participation of these functional groups in the synthesis process. The FTIR results obviously demonstrate that the surface structure of the ZnO synthesized by fruit, seed and pulp extracts are different, and this influences the surface layer of Zn-O bonds. This might affect the properties of ZnO-NPs that depend mainly on the surface structures.

### 2.2. Analysis of Optical Properties

**Figure 6:**
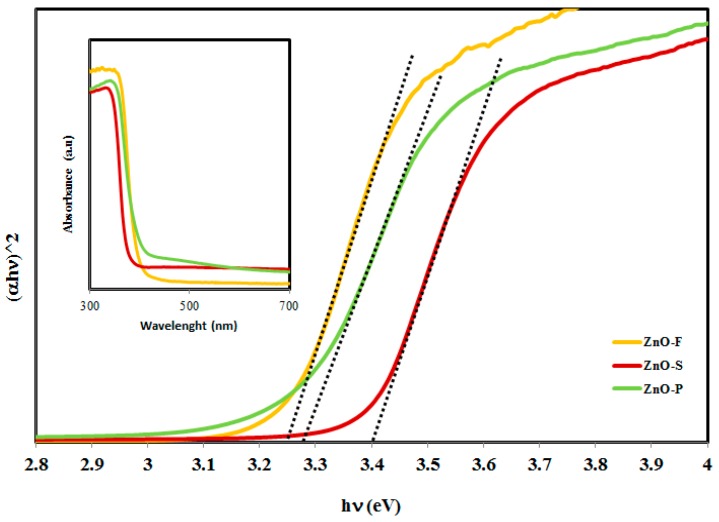
Absorption edge (inset) and band gap of the prepared ZnO-NPs.

The UV-vis spectra of ZnO-NPs bio-synthesized by use of fruit, seed and pulp of *C. colocynthis* are shown in the inset of Figure 6. The relevant increase in the absorption at wavelengths more than 350 nm can be assigned to the direct band-gap of ZnO due to the electron transitions from the valence band to the conduction band (O2p-Zn3d) [36]. A redshift in the absorption edge was seen for the ZnO fruit (ZnO-F) and ZnO pulp (ZnO-P) compared to ZnO seed (ZnO-S). This might be owing to changes in its particle size, morphology and surface microstructure. Furthermore, the direct band-gap energies estimated from a plot of (αhν)^2^ versus the photo energy (*hν*) consistent with the Kubelka-Munk model shown in Figure 6 were 3.40, 3.28 and 3.25 eV for the ZnO-NPs, synthesized by seed, pulp, and fruit extracts, respectively. Such a decrease in the ZnO band-gap energy is well consistent with the corresponding redshift seen in the absorption edge noted above. ZnO in the form of bulk has a band gap of ∼3.20 eV, which is in the close UV region. However, when ZnO is synthesized in nano-size range, the band gap increases owing to the quantum confinement effect, and this describes the larger band gap of the ZnO-NP with smaller size.

### 2.3. Analysis on Colloidal Properties

In this study, zeta potential and hydrodynamic size were measured by DLS. The zeta potential for colloidal suspensions of ZnO-NPs samples were found at −25.60, −19.5 and −15.3 mV for ZnO-F, ZnO-P and ZnO-S, respectively. From the zeta potential values, it is suggested that the NPs were stable and warped with anionic organic phases and responsible for electrostatic stabilization [37]. FTIR result has already revealed that the surface structure of the synthesized ZnO samples by fruit, seed and pulp are somewhat different. This difference is expected to change the net surface charge density and their inter particle interactions.

Nanoparticles generally suffer from agglomeration when dispersed in solutions, and this behavior has a major effect on the reactivity and response of nanomaterials upon contact to various cells or organisms [38]. Hence, the hydrodynamic sizes of the ZnO-NPs dispersed in nanopure water were recorded at physiological pH. The hydrodynamic sizes of the ZnO-NPs produced by seed, pulp and fruit were 1050 ± 80 nm, 860 ± 80 nm, and 750 ± 80 nm, respectively. Based on the Derjaguin-Landau-Verwey-Overbeek (DLVO) model, aggregation of uncovered nanoparticles depends on the repulsive forces arising from electrostatic and the van der Waals forces of attraction. Because the surface charges of nanoparticles affect the electrostatic repulsive interaction, nanoparticles with higher zeta potential value will usually decrease hydrodynamic size. In addition, the polydispersity indices (PDIs) of ZnO-NPs obtained from seed, pulp and fruit are 0.369, 0.385 and 0.407 (μ_2_/Г^2^), respectively (Table 2). These results show that the nanoparticles have an intermediate, moderately polydispersed distribution type, where the distribution is neither very polydispersed, or broad, nor in any sense narrow.

### 2.4. In Vitro Cytotoxicity Study

Cytotoxicity is a new property exposed by various materials, including ZnO, when produced with the nano-size dimensions. According to this, we attempted to investigate and compare the cytotoxicity of ZnO-NPs synthesized by seed, pulp and fruit extracts on 3T3 cells to determine if the differences in the size, shape and surface structures of these three ZnO-NP samples have an important effect on their interaction with these cells. To evaluate the cell viability of 3T3 cells treated with each of the ZnO-NP samples, different ZnO-NP concentrations of 0, 0.05, 0.1, 0.15, 0.2, 0.3 and 0.4 mg/mL were employed, and the cell viability was determined after 72 h of treatment.

**Figure 7:**
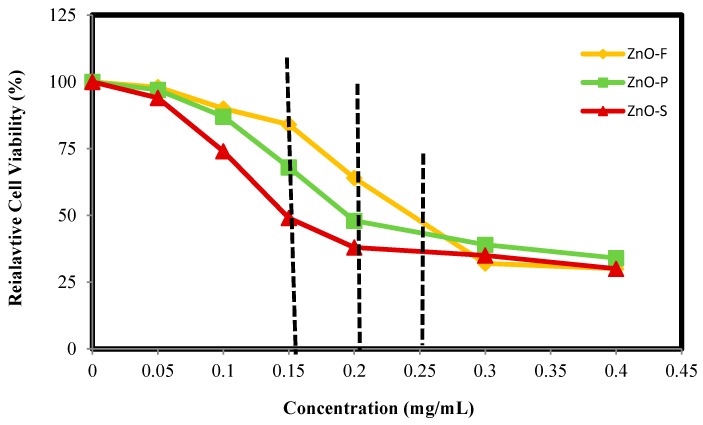
Cytotoxic effect of ZnO-NPs samples on the growth of 3T3 cells.

The cell viability data of ZnO-NP samples are shown in Figure 7. It displays a slow decrease in cell viability with increasing concentration of ZnO-NP for all the ZnO samples. However, the ZnO-S displayed a slightly faster reduction in the cell viability with increasing nanoparticles concentration compared to ZnO-P and ZnO-F, so that the half maximal inhibitory concentration IC50 of ZnO-S was 0.160 mg/mL, while those of ZnO-P and ZnO-F were 0.210 and 0.258 mg/mL, respectively. The ZnO-S sample showed about ~1.3 and ~1.6 times higher IC50 compared to those of the ZnO-P and ZnO-F samples respectively, suggesting that ZnO-S NP is more toxic than the ZnO-P and ZnO-F nanoparticles. The cytotoxicity of synthesized ZnO-NPs can be related to their size and morphology. The smaller size nanoparticles can penetrate easily into cell membrane and caused more cytotoxicity compared to larger size nanoparticles [39]. This result is in agreement with previous findings [40,41] which showed that a variety of factors, such as a nanoparticle's shape, size, surface charge effect on cytotoxicity of nanoparticles.

### 2.5. 1,1-Diphenyl-2-picrylhydrazyl (DPPH) Radical Scavenging Activity

**Figure 8:**
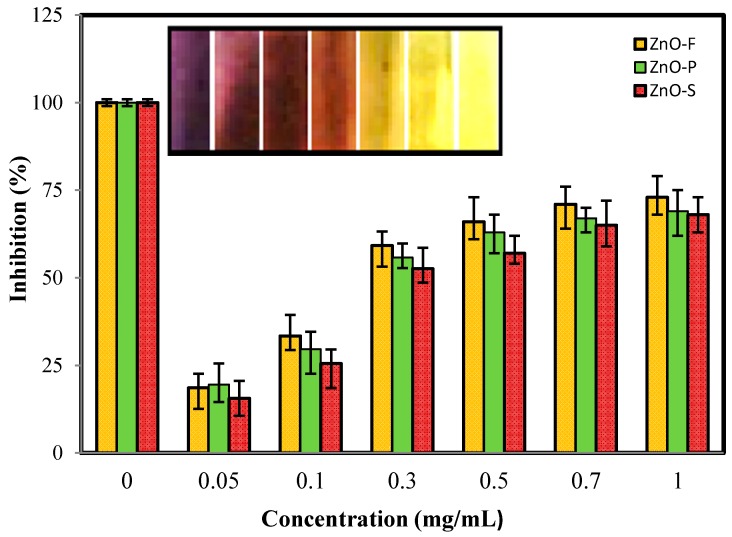
Scavenging capacity of the prepared ZnO samples on 1,1-Diphenyl-2-picrylhydrazyl (DPPH) (DPPH) free radicals and color changes of DPPH with different concentration of ZnO-NPs (inset).

The free radical scavenging activity of ZnO-NPs were examined by DPPH scavenging and are shown in Figure 8. DPPH is a stable free radical and shows a typical absorption peak at 517 nm. The decrease in absorption is taken as a quantity of the level of radical scavenging. The change of color from dark purple to light yellow is proportional to reduction of DPPH and conversion to 1,1-diphenyl-2-picryl hydrazine with decolorization (see inset in Figure 8). The DPPH activity of the ZnO-NPs was found to increase in a dose-dependent manner. Scavenging of DPPH radicals was found to be increasing as the concentration of the ZnO-NPs samples increased. Among, these nanoparticles the ZnO-F sample displayed a higher scavenging of DPPH radicals with increasing concentration than that of the ZnO-S and ZnO-P. Considering the more negative surface charge of ZnO-F compared to ZnO-S and ZnO-P, it appears that these ZnO-F NPs display a high tendency to interact with and reduce DPPH. The all-ZnO nanoparticles were proved to be potent at inhibiting the DPPH free radical scavenging activity with IC50 value of 0.22, 0.26 and 0.29 mg/mL for ZnO-F, ZnO-P and ZnO-S NPs, respectively.

### 2.6. Antimicrobial Activity

**Figure 9:**
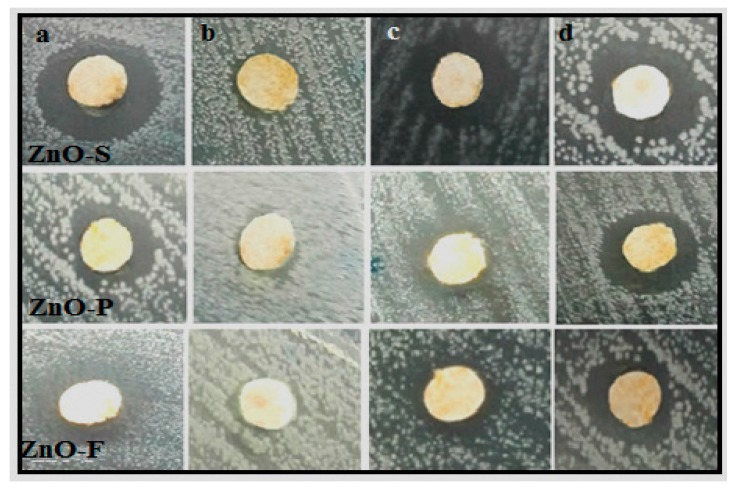
Inhibition zone of synthesized ZnO-NPs against (**a**) *B. subtilis*; (**b**) *MRSA*; (**c**) *P. aeruginosa* and (**d**) *E. coli* pathogens.

In this study, the antimicrobial activity of ZnO-NPs synthesized using new bio-fuels was evaluated. In this analysis, the ZnO-NPs showed antimicrobial activity against a range of different bacteria (Table 3, Figure 9). The ZnO-S nanoparticles showed the highest antibacterial activity compared to the two others ZnO-NPs samples. The reason for enhancement is probably due to the small size of ZnO-S which is more likely to penetrate into the cell membrane to kill the bacteria. The zone of inhibition in the case of ZnO-S nanoparticles for each bacterium were determined to be approximately 14.3 ± 0.51, 6.8 ± 0.36, 12.2 ± 0.27 and 13.4 ± 0.47mm, respectively, for *B. subtilis*, Methicillin-resistant *Staphylococcus aurous* (MRSA), *P. aeruginosa* and *E. coli*. The highest antimicrobial activity was observed against *B.subtilis*, *P. aeruginosa* and *E. coli*, while a lower activity was found against MRSA. This difference can be related to the structural and chemical composition of the cell membrane of micro-organisms. These findings are consistent with earlier studies that studied the antimicrobial activity of ZnO against *B. subtilis*, *P. aeruginosa* [42] and *E. coli* [43,44].

## 3. Materials and Methods

### 3.1. Preparation of the *C. colocynthis* Extracts

Mature fruits of *C. colocynthis* were collected from Koohzar district, Khuzestan, Iran in April. The whole fruit were oven-dried at 45 °C for 48 h, and then fruit, seed and pulp separately milled to fine powder. About 5 g from each fine powder was dispersed in 50 mL of distilled water and magnetically stirred for 30 min at 100 °C. The extracts were filtered and centrifuged to eliminate any bulks and kept at −20 °C before use.

### 3.2. Synthesis of ZnO-NPs

**Figure 10:**
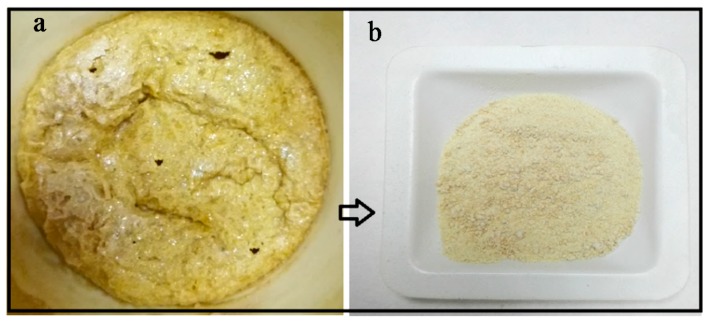
Photograph formation of (**a**) foam and prepared (**b**) ZnO-NPs.

In a typical synthesis, 6 g of Zn(NO_3_)_2_ 6H_2_O was dissolved in 2 mL distilled water in a porcelain crucible and then thoroughly mixed with 5 mL of the extract (fruit, seed or pulp) under stirring and finally was placed under microwave irradiation at 340 W. After boiling and evaporating, the mixture quickly foamed up, and released gases. The whole process was completed in 8 min and the ZnO-NPs were left as residue (Figure 10). The ZnO-NPs samples synthesized by fruit, seed and pulp extracts called ZnO-F, ZnO-S and ZnO-P, respectively.

### 3.3. Characterization of ZnO-NPs

The crystal structure of ZnO-NPs were analyzed by PXRD (Philips, X’pert, Cu Kα) in the 2θ range between (2–80°) at room temperature. FTIR spectra of the samples were recorded by FTIR spectrometer (Perkin-Elmer 1725X, Waltham, MA, USA), with a resolution of 4.0 cm^−1^ at wavenumber range from 400 to 4000 cm^−1^. The UV-Visible absorption of powder samples were examined by UV-Vis spectrophotometer (a Lambda 25-Perkin Elmer, Waltham, MA, USA) in the range of 200–800 nm. Transmission electron microscope (HITACHI H-700, Tokyo, Japan) with an acceleration voltage of 120 kV was applied to study size and morphology of the ZnO-NPs. TEM samples were prepared by depositing a few drops of the sample suspension on a copper grid followed with drying at room temperature. The morphology of samples were further observed using FESEM (JSM-6360LA, Eindhoven, The Netherlands). The small amounts of powder samples were mounted on a metal stub using carbon tape and then gold-coated using a sputter coater. The particle sizes and the zeta potentials of ZnO-NPs were analyzed by photon correlation spectroscopy and laser Doppler anemometry, respectively, with a Zetasizer, Nano ZS (Malvern Instruments Ltd., Malvern, UK) at room temperature.

### 3.4. MTT Assay for Cell Viability

The in vitro cytotoxicity of ZnO-NPs samples was assessed by the method using 3-(4,5-dimethylthiazol-2-yl)-2,5-dephenyl-tetrazolium bromide (MTT) assay. Briefly, 3T3 cells were seeded at a density of 2 × 105 cells/mL in 96-well microplates and incubated for 24 h. Subsequently, the cells were treated with the various concentrations of samples in the presence of 10% FBS for 24 h. The samples were suspended separately in a stock solution at 5 μg/mL in a solution of dimethyl sulfoxide (DMSO)/double distilled water. After 24 h of incubation, 20 μL of 5 mg/mL MTT in the PBS buffer was added to each well, and the cells were incubated for another 4 h at 37 °C. The 100 μL of DMSO was added to dissolve the formazan crystal formed by live cells. Optical absorbance was measured at 570 nm. Cell viability was calculated as the percentage of absorbent compared to control. The 50% inhibitory concentration (IC_50_) value, defined as the amount of sample that inhibits 50% of cell growth, was calculated from the concentration–response curves.

### 3.5. DPPH Radical Scavenging Assay

Bio-synthesized ZnO-NPs samples were assessed for the scavenging effect on DPPH radical according to the method of Blois [45]. Different concentrations (0.05–1 mg/mL) of the samples were added, in equal volume (3 mL), to 0.1 mM methanolic DPPH solution. The reaction mixture was incubated for 30 min at room temperature under vigorous shaking and the absorbance was recorded at 517 nm. Glutation (GSH) was used as control. All determinations were performed in triplicate. The DPPH radical scavenging activity (RSA) was expressed in percentage of inhibition using the following formula.

(1) $$\%\mathrm{RSA}=[1-\frac{A_{Sample}}{A_{Control}}]\times 100$$

### 3.6. Antimicrobial Assessment

In the present study, in vitro antimicrobial activities of prepared ZnO samples towards gram-positive (*Bacillus subtilis* (*B. subtilis*) B29 and Methicillin-resistant *Staphylococcus aurous* (MRSA)) and gram-negative (*Peseudomonas aeruginosa* (*P. aeruginosa*) ATCC 15442 and *Escherichia coli* (*E. coli*) E266) pathogens were performed through a disc-diffusion assay. Briefly, the bacteria were grown overnight in nutrient broth. The bacterial inoculum was standardized to 0.5 MF units, meaning that approximately 10^8^ colony-forming units of each bacterium were inoculated onto a plate. Previously prepared samples impregnated discs (6 mm) at the various concentrations were placed aseptically on plates inoculated with bacteria and incubated at 37 °C for 24 h. After incubation, the zone of whole inhibition was measured. All tests were replicated three times.

## 4. Conclusions

We have developed a simple, rapid and eco-friendly process to prepare ZnO nanocrystals using bio-fuels of fruit, seed and pulp extracts of *C. colocynthis* through microwave-assisted combustion. The bio-fuels could effectively mediate the nucleation and growth of ZnO nanoparticles under microwave radiation. Results reveal that the bio-fuels significantly affect the morphology of ZnO. Flower-like, hexagonal, and block-shaped nanostructures were produced by fruit, seed and pulp, respectively. The particle size of nanoparticles was varied between ~27 to ~85 nm. The ZnO samples with different shapes, sizes and chemical surfaces revealed some differences in biological activities. The as-synthesized nanoparticles with low toxicity exhibited desirable antioxidant and antimicrobial activities. This study showed that different parts of *C. colocynthis* fruit are suitable fuels to prepare ZnO-NPs and could be extended to synthesize other related metal oxide materials by microwave-assisted combustion.

## Figures and Tables

**Table 1:** The lattice parameters of the synthesized ZnO-NPs.

Sample	2θ ± 0.1	*h k l*	Structure	Lattice Parameter (nm)	V (A°)^3^	*cos*φ
ZnO-F	67.96	1 1 2	wurtzite	*a* = 0.3222, *c/a* = 1.599	48.17	0.8480
	69.10	2 0 1				
ZnO-S	68.08	1 1 2	wurtzite	*a* = 0.3248, *c/a* = 1.592	47.88	0.7193
	69.18	2 0 1				
ZnO-P	67.92	1 1 2	wurtzite	*a* = 0.3249, *c/a* = 1.601	47.62	0.7180
	69.15	2 0 1				

**Table 2:** Colloidal properties of ZnO-S, ZnO-P and ZnO-F.

Sample	Zeta Potential (mV)	Hydrodynamic Size (nm)	Polydispersity Indices (μ_2_/Г^2^)
ZnO-S	−15.3 ± 2	1050 ± 80	0.369
ZnO-P	−19.5 ± 2	860 ± 80	0.385
ZnO-F	−25.60 ± 2	750 ± 80	0.407

**Table 3:** Mean inhibition zone (mm) of synthesized ZnO-NPs against different pathogens.

Pathogens	ZnO-S	ZnO-P	ZnO-F
*B. subtilis*	14.3 ± 0.51	11.2 ± 0.25	12.2 ± 0.24
MR*SA*	6.8 ± 0.36	6.9 ± 0.12	6.8 ± 0.39
*P. aeruginosa*	12.2 ± 0.27	10.7 ± 0.49	10.4 ± 0.49
*E. coli*	13.4 ± 0.47	11.8 ± 0.28	10.8 ± 0.10
